# Impact of Large Language Model Assistance on Radiologists’ Diagnostic Performance for Brain Tumors by Experience Level

**DOI:** 10.3390/jcm15041673

**Published:** 2026-02-23

**Authors:** Chae Won Song, Byung Hyun Baek, Seul Kee Kim, Woong Yoon, Yun Young Lee, Ilwoo Park, Jae Hyun Park, Seol Bin Park, In Woo Choi

**Affiliations:** 1 Department of Radiology, Chonnam National University Hospital, Gwangju 61469, Republic of Korea; bambi7474@naver.com (C.W.S.); radyoon@jnu.ac.kr (W.Y.); yunyoung0219@gmail.com (Y.Y.L.); ipark@jnu.ac.kr (I.P.); jenny_94@naver.com (J.H.P.); dma0430@naver.com (S.B.P.); 2 Department of Radiology, Chonnam National University Medical School, Gwangju 61469, Republic of Korea; cnu.radiology0@gmail.com; 3 Department of Radiology, Chonnam National University Hwasun Hospital, Hwasun 58128, Republic of Korea; 4 Department of Data Science, Chonnam National University, Gwangju 61180, Republic of Korea; 5 Department of Artificial Intelligence Convergence, Chonnam National University, Gwangju 61180, Republic of Korea

**Keywords:** large language models, artificial intelligence, brain tumors, magnetic resonance imaging, Chat Generative Pre-trained Transformer (ChatGPT)-4o, Claude 3.5 Sonnet

## Abstract

**Background**: Large language models (LLMs) may assist radiologists in interpreting brain tumor MRI. We compared the diagnostic accuracy of ChatGPT-4o and Claude 3.5 Sonnet with that of board-certified radiologists and trainees, and evaluated whether LLM assistance could enhance diagnostic performance. **Methods**: A total of 127 histologically confirmed brain tumor cases were included. Two LLMs analyzed representative MRI images together with structured radiologic reports, whereas two board-certified radiologists and three trainees reviewed representative images with basic demographic information only. All participants generated up to three differential diagnoses per case. The accuracy of the primary diagnosis and the accuracy of the top-three differential diagnoses were calculated and compared. Following the initial readings, LLM-generated differential diagnoses were provided to the readers, and their post-assistance diagnostic performance was re-evaluated. **Results**: Claude 3.5 Sonnet achieved a primary diagnostic accuracy of 50.4% and a top-three differential accuracy of 85.0%, comparable to ChatGPT-4o (44.9% and 82.7%, respectively). Radiologists demonstrated a higher primary diagnostic accuracy (69.3%, *p* < 0.001) compared to LLMs, but a similar top-three differential accuracy (80.7%). In contrast, trainees showed a primary diagnostic accuracy (48.0%) comparable to LLMs, but a lower top-three differential accuracy (62.5%) than LLMs. With LLM assistance, radiologists exhibited a significant improvement in the top-three differential accuracy (from 80.7% to 90.2%, *p* < 0.001), and trainees showed significant improvements in both the primary and top-three differential accuracy (from 48.0% to 58.8%, *p* < 0.001, and from 62.5% to 81.1%, *p* < 0.001, respectively). **Conclusion**: LLMs demonstrated the ability to expand differential diagnostic considerations when operating on structured imaging inputs. LLM assistance was associated with improved trainee performance in this constrained experimental setting. These findings should be interpreted cautiously and require validation under balanced input conditions and clinically realistic workflows.

## 1. Introduction

Artificial intelligence (AI) has rapidly expanded within healthcare, particularly in diagnostic radiology. Large language models (LLMs), such as ChatGPT and Claude, have demonstrated their capacity to process vast amounts of data and provide decision support in complex clinical scenarios. These models are being explored for their potential to enhance diagnostic accuracy, improve efficiency, and reduce radiologist workload, particularly in high-demand specialties such as neuroradiology [1,2].

Recent studies have demonstrated the potential of LLMs to improve diagnostic workflows by assisting with differential diagnosis generation and reducing diagnostic variability among radiologists. For example, fine-tuning LLMs through effective prompt engineering has shown promising results in improving diagnostic accuracy, especially in neuroradiology [3,4]. One significant advantage of LLMs lies in their ability to synthesize and process a vast amount of medical literature, which enables radiologists to make more informed decisions [5].

Earlier studies evaluated the diagnostic performance of LLMs using “Case of the Week” cases from the American Journal of Neuroradiology, “Diagnosis Please” cases from Radiology, or archived cases from “Neuroradiology: A Core Review,” along with brief patient histories [6,7,8,9,10,11]. These cases are typically designed as challenging quiz-style scenarios for expert radiologists and provide only a limited number of images rather than full clinical magnetic resonance imaging (MRI) datasets. As such, they do not accurately reflect real-world clinical practice. This likely contributed to the low diagnostic accuracy observed in these studies. As a result, LLMs consistently underperformed compared with radiologists, with Horiuchi et al. reporting an accuracy as low as 16% for central nervous system (CNS) tumors [6]. Brain tumors are particularly challenging to diagnose due to their wide variety of subtypes and subtle, overlapping MRI features.

More recent research has attempted to enhance LLM performance in neuroradiology. Wada et al. demonstrated that prompt engineering strategies can significantly improve the diagnostic accuracy of GPT-4 Turbo [7]. Furthermore, studies using structured MRI reports of brain tumors have shown that GPT-4-based models can achieve diagnostic performance comparable to neuroradiologists in differentiating tumor types [5,12]. In addition, several comparative studies in the neuroradiology field have recently evaluated the performance of multiple state-of-the-art LLMs, collectively demonstrating a gradual improvement in diagnostic accuracy as model architectures and prompting strategies continue to advance [12,13,14].

Therefore, this study first compared the diagnostic performance of two advanced LLMs—ChatGPT-4o (OpenAI, San Francisco, CA, USA) and Claude 3.5 Sonnet (Anthropic, San Francisco, CA, USA)—and then evaluated the better-performing model against radiologists with varying levels of experience. We further assessed whether assistance from this LLM improved radiologists’ diagnostic performance.

## 2. Materials and Methods

### 2.1. Patient Selection and Case Distribution

A total of 127 histologically confirmed brain tumor cases were selected from patients who underwent surgery or biopsy at Chonnam National University Hwasun Hospital between 2014 and 2023. The study protocol was approved by the Institutional Review Board of Chonnam National University Hwasun Hospital (approval number: CNUHH-2024-176), and the requirement for informed consent was waived due to the retrospective nature of the study.

The cases were selected to include a broad spectrum of brain tumor subtypes, ensuring representation of both common and rare intra-axial brain tumors. The average age of the patients was 40.3 years (range, 1 to 87 years), with a nearly equal gender distribution (62 males and 65 females). Tumor subtypes were categorized based on their incidence rates in both adult and pediatric populations. High-incidence tumors, such as glioblastoma and primary CNS lymphoma, were represented by 15 cases each, selected from the most recent diagnoses within the study period. For moderate-incidence tumors, including ependymoma and pilocytic astrocytoma, 10 cases of each were included. Rare tumor types, such as ganglioglioma and dysembryoplastic neuroepithelial tumor (DNET), were fully represented, with fewer than 10 cases included for these subtypes based on availability within the 10-year study period.

The case selection process ensured a diverse and clinically representative set that encompassed a wide range of diagnostic challenges typically encountered in clinical practice. The specific distribution of tumor subtypes and the corresponding number of cases for each subtype are detailed in Table 1.

### 2.2. Imaging Data and Structured Report Preparation

For each case, we selected key MRI sequences routinely used in brain tumor diagnosis, including T1-weighted images (pre- and post-contrast), T2-weighted images, fluid-attenuated inversion recovery (FLAIR), and diffusion-weighted imaging (DWI). In cases where advanced MRI sequences such as susceptibility-weighted imaging (SWI), perfusion-weighted imaging (PWI), or MR spectroscopy were available, one or two representative images from these sequences were also included in the diagnostic set. These imaging modalities were chosen based on their ability to highlight key diagnostic features of brain tumors, such as enhancement patterns (T1 post-contrast), tumor size, shape, and edema (FLAIR), and diffusion restriction (DWI), which are essential for accurate diagnosis.

**Figure 1:**
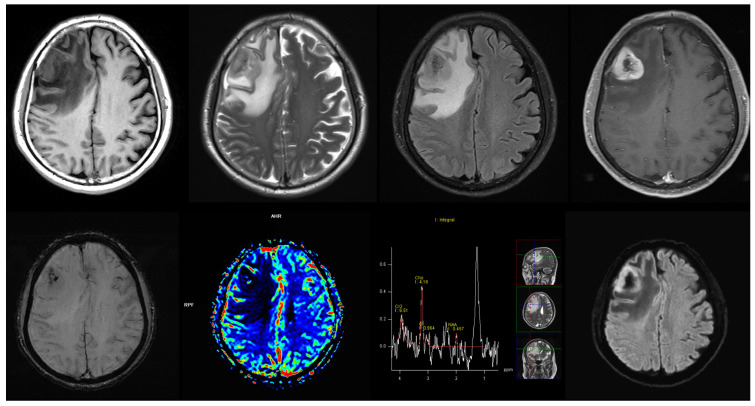
A representative example of multimodal magnetic resonance imaging data from a patient with atypical primary CNS lymphoma. The lesion in the right frontal lobe demonstrated intratumoral necrosis and hemorrhagic components, imaging features that can mimic metastasis or glioblastoma. However, pathology confirmed the diagnosis of lymphoma.

All imaging data were anonymized to ensure patient confidentiality and subsequently compiled into a single PowerPoint slide for each case. This slide, which included representative images from the selected sequences, was provided to both the LLMs and radiologists for evaluation. The case selection and the imaging data preparation were carried out by an expert neuroradiologist (initials: W.Y., with 25 years of experience). Figure 1 is a representative example of this slide.

**Figure 2:**
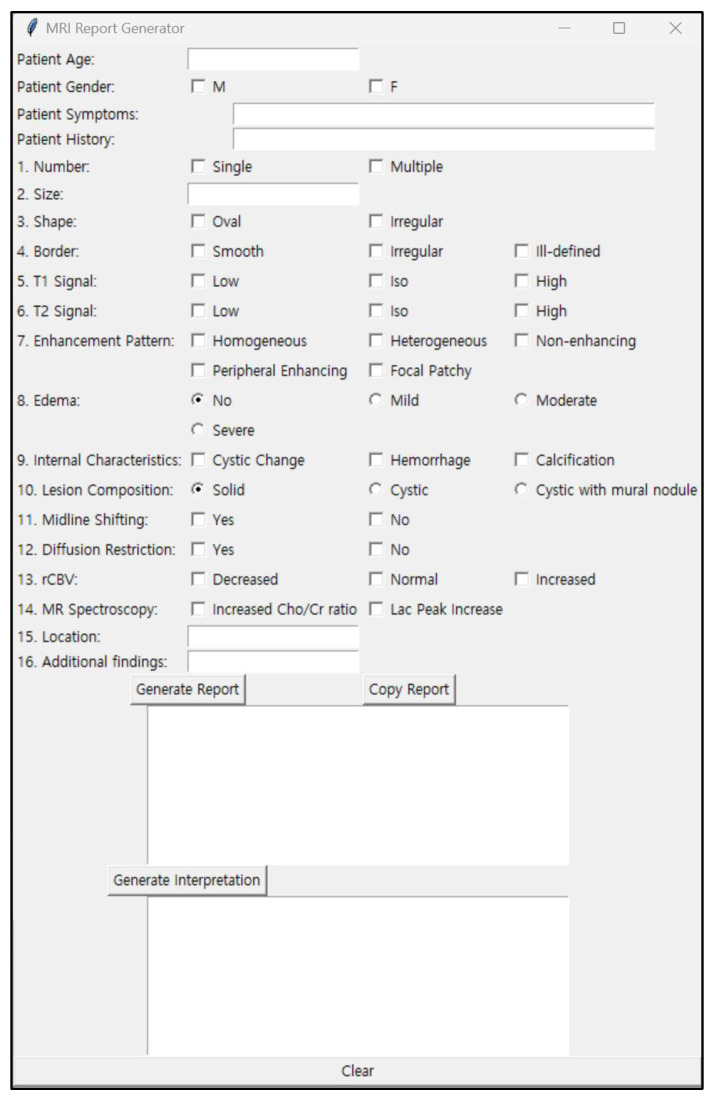
MRI structured report generator interface (developed using Python).

To standardize the reporting of imaging findings and facilitate consistent and detailed documentation across all cases, we developed a structured report generator using Python (version 3.8) (Figure 2). A structured report form was created by an expert neuroradiologist (initials: Y.Y.L., with 10 years of experience), who was blinded to the clinical details of each case, to ensure standardized and objective evaluation of imaging features. This tool allowed the systematic input of key diagnostic features, such as tumor size, shape, enhancement patterns, presence of edema, location, and other internal characteristics (e.g., cystic changes, hemorrhage, or calcification). Additional clinical information, including patient age and gender, was also incorporated into the structured report, providing a comprehensive input for diagnostic analysis.

### 2.3. Large Language Models (LLMs) and Diagnostic Task

In this study, two of the most advanced LLMs available at the time—ChatGPT-4o (OpenAI, San Francisco, CA, USA) and Claude 3.5 Sonnet (Anthropic, San Francisco, CA, USA)—were evaluated for their diagnostic performance in analyzing brain tumor findings. These models were selected as state-of-the-art, widely used LLMs available at the time of the study, enabling evaluation of contemporary performance in a clinically relevant diagnostic task.

**Figure 3:**
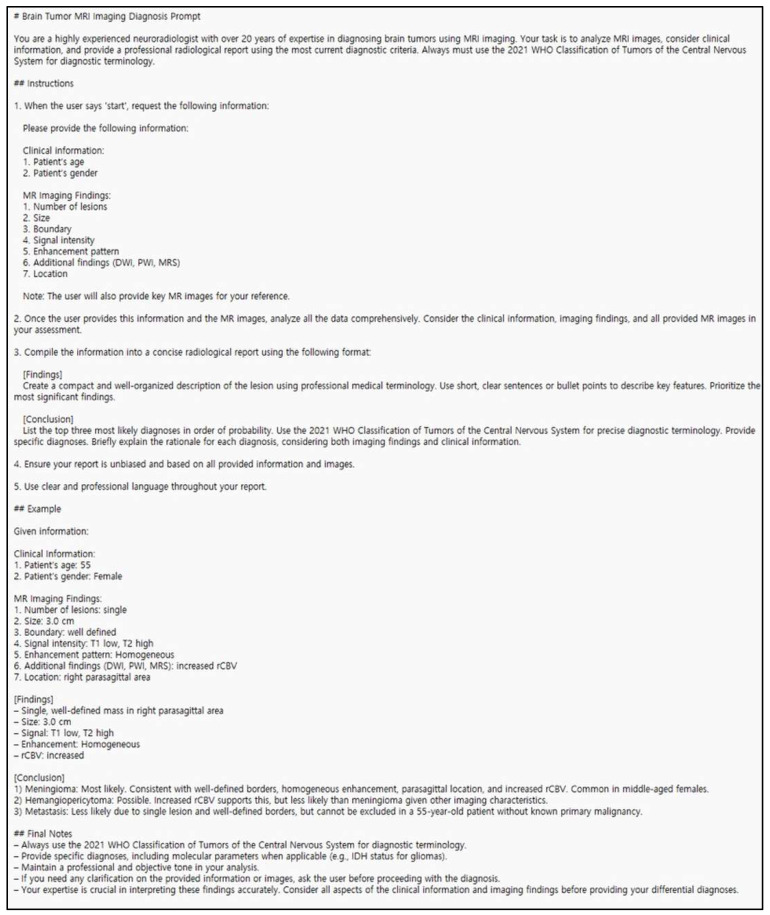
Prompt used for ChatGPT-based brain tumor MR imaging diagnosis in this study.

Each LLM was tasked with generating up to three differential diagnoses for the 127 histologically confirmed brain tumor cases based on the provided MRI images (compiled into a single PowerPoint slide) and the accompanying structured radiologic report. The interaction process was standardized through the use of a custom-designed, identical prompt for both models to ensure consistency in task execution and output. The prompt simulated the reasoning process of an experienced neuroradiologist, requesting the synthesis of clinical information and MRI findings to produce a structured radiologic report that included up to three differential diagnoses based on the 2021 WHO classification of Tumors of the Central Nervous System. Full details of the prompt are provided in Figure 3.

**Figure 4:**
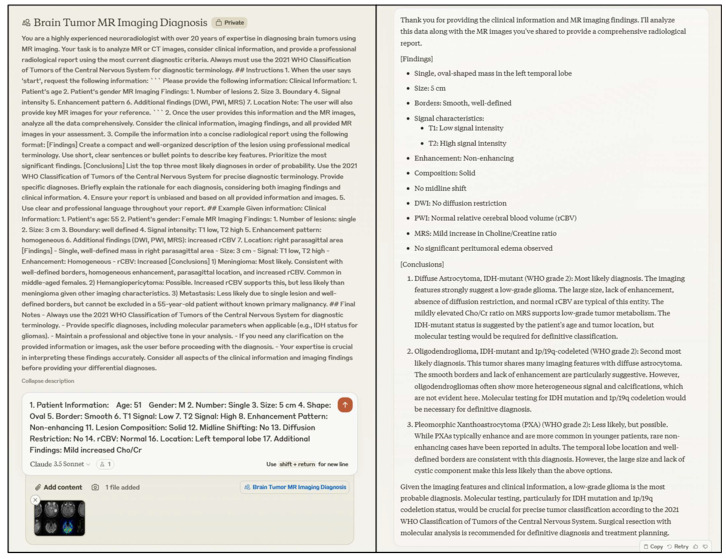
Representative interaction between Claude 3.5 Sonnet and structured imaging findings provided by a radiologist for generating differential diagnoses.

Once this prompt was delivered, both ChatGPT-4o and Claude 3.5 Sonnet analyzed the anonymized MRI images compiled into a single PowerPoint slide, along with the structured radiologic reports. The LLMs were configured not to store any conversation data, ensuring privacy and security compliance. Each LLM produced a concise report detailing the radiological findings and offering three differential diagnoses in order of likelihood. Figure 4 illustrates the structured interaction with Claude 3.5 Sonnet for generating differential diagnoses.

The LLM with the superior diagnostic performance—defined by accuracy in both primary diagnosis and top-three differential diagnosis accuracy—was selected for further comparison and assistance tasks with the radiologists. In cases where the best-performing model differed between the two metrics, the model with higher accuracy for each respective metric was used.

### 2.4. Radiologist Evaluation and LLM Assistance

Five radiologists participated in the study: Two board-certified neuroradiologists (initials: S.K.K. and B.H.B., with 15 and 10 years of experience, respectively) and three trainee radiologists (initials: S.B.P., J.H.P., and E.J.K., with 3, 3, and 2.5 years of training, respectively). The radiologists were provided with the same key MRI images as the LLMs, presented in a single PowerPoint slide, but without access to the structured reports or any clinical information beyond the patient’s age and gender. Each radiologist was required to generate up to three differential diagnoses for each case.

**Figure 5:**
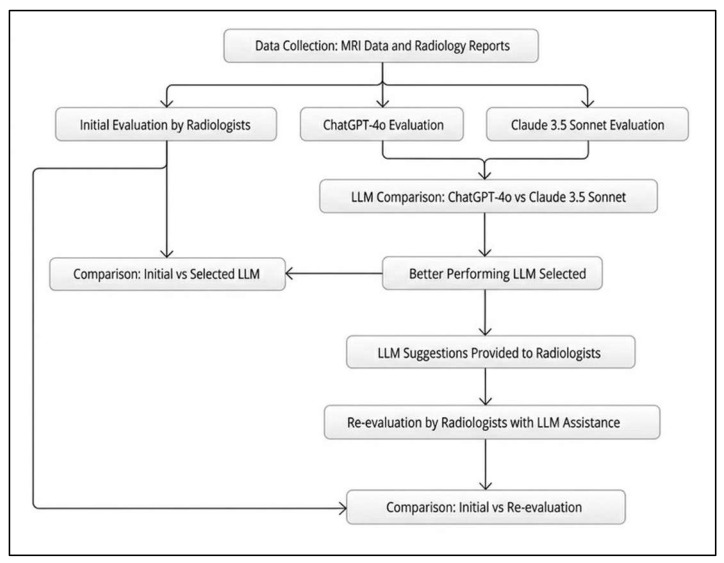
Diagnostic Workflow for baseline and LLM-assisted readings. Radiologists first reviewed a single PowerPoint slide of representative MRI images for each case together with patient age and sex and generated up to three differential diagnoses (baseline). Separately, the LLM analyzed the same image slide plus the structured MRI report and generated up to three differential diagnoses. For the assisted reading, radiologists were provided with the LLM-generated differential diagnosis list and then revised their diagnoses, which were recorded for post-assistance evaluation.

Following the initial diagnostic session, the differential diagnoses produced by the better-performing LLM were shared with the radiologists. The radiologists were then asked to re-evaluate the cases and were given an opportunity to revise their diagnoses based on the LLM’s suggestions, enabling an assessment of potential diagnostic improvements through AI assistance (Figure 5).

### 2.5. Statistical Analysis

Statistical analyses were conducted using Python (version 3.8) with the scikit-learn (version 0.24.2) and SciPy (version 1.7.1) packages. The following analyses were performed:

1. Primary diagnosis accuracy: Compared using McNemar’s test, with odds ratios (OR) and 95% confidence intervals (CI) calculated to quantify the likelihood of correct diagnoses between the models and radiologists.
2. Top-three differential diagnosis accuracy: Evaluated using Cochran’s Q test, followed by post hoc pairwise comparisons with Bonferroni correction for multiple comparisons.
3. Changes in radiologist performance after LLM assistance: Given the small number of readers, we used the Wilcoxon signed-rank test for within-reader comparisons. To account for multiple hypothesis testing across reader groups and diagnostic endpoints, Holm–Bonferroni correction was applied to control the family-wise error rate.

All statistical analyses were conducted at a significance level of *p* < 0.05. 

## 3. Results

### 3.1. Diagnostic Performance of Large Language Models (LLMs)

The analysis of 127 histologically confirmed brain tumor cases demonstrated comparable diagnostic performance between ChatGPT-4o and Claude 3.5 Sonnet. ChatGPT-4o achieved a primary diagnosis accuracy of 44.9% (95% CI: 36.3–53.7%), while Claude 3.5 Sonnet reached 50.4% (95% CI: 41.6–59.1%). McNemar’s test showed no statistically significant difference between the two models (*p* = 0.296). For top-three differential diagnosis accuracy, ChatGPT-4o achieved 82.7% (95% CI: 75.0–88.6%) compared to Claude 3.5 Sonnet’s 85.0% (95% CI: 77.6–90.5%). Cochran’s Q test similarly showed no significant difference between the models (*p* = 0.623).

Based on this slightly superior performance in both metrics, Claude 3.5 Sonnet was selected as the benchmark LLM for further comparison against radiologists and subsequent integration into the assistance analyses. Although the performance differences between the models were not statistically significant, Claude 3.5 Sonnet consistently demonstrated slightly higher diagnostic accuracy across both evaluation metrics, supporting its selection as the benchmark model.

### 3.2. Comparison of LLM and Radiologist Performance

The diagnostic performance of Claude 3.5 Sonnet was evaluated against both board-certified radiologists (*n* = 2) and trainee radiologists (*n* = 3). The results are summarized in Table 2. Claude 3.5 Sonnet achieved a primary diagnosis accuracy of 50.4% (95% CI: 41.6–59.1%) and a top-three differential diagnosis accuracy of 85.0% (95% CI: 77.6–90.5%). In comparison, board-certified radiologists achieved a primary diagnosis accuracy of 69.3% (95% CI: 60.8–76.6%) and a top-three differential diagnosis accuracy of 80.7% (95% CI: 72.6–86.3%). Trainee radiologists demonstrated a primary diagnosis accuracy of 48.0% (95% CI: 39.5–56.7%) and a top-three differential diagnosis accuracy of 62.5% (95% CI: 53.5–70.2%).

Statistical analysis revealed that the primary diagnosis accuracy of Claude 3.5 Sonnet was significantly lower than that of board-certified radiologists (*p* < 0.001), but comparable to trainee radiologists (*p* = 0.54). For top-three differential diagnosis accuracy, Claude 3.5 Sonnet outperformed trainee radiologists (*p* < 0.001) but showed no statistically significant difference when compared to board-certified radiologists (*p* = 0.194).

### 3.3. Impact of LLM Assistance on Radiologist Performance

The introduction of LLM-generated differential diagnoses into the radiologists’ workflow significantly improved diagnostic accuracy, particularly among trainee radiologists (Table 3).

Among board-certified radiologists, primary diagnosis accuracy increased modestly from 69.3% to 71.3% following LLM assistance (*p* = 0.166), while top-three differential diagnosis accuracy improved significantly from 80.7% to 90.2% (*p* < 0.001). For trainee radiologists, the improvements were more pronounced: Primary diagnosis accuracy increased from 48.0% to 58.8% (*p* < 0.001), and top-three differential diagnosis accuracy rose from 62.5% to 81.1% (*p* < 0.001). Notably, the post-LLM assistance performance of trainee radiologists (81.1%) essentially matched the baseline performance of board-certified radiologists (80.7%) in terms of top-three differential diagnosis accuracy.

### 3.4. Subgroup Analysis by Tumor Type

A subgroup analysis of diagnostic performance across different tumor types revealed variability in LLM accuracy. Both LLMs demonstrated high accuracy in diagnosing glioblastoma, with primary diagnosis accuracy nearing 90%. For astrocytoma (grades II and III), the accuracy averaged around 80% for both models. However, performance was substantially lower for more complex tumor types, such as primary CNS lymphoma, where primary diagnosis accuracy dropped to 40%.

Overall, while the LLMs showed robust accuracy for more common brain tumor subtypes, their performance was more limited for rarer or complex tumor presentations. This highlights the need for further refinement in LLM capabilities when applied to challenging and less frequently encountered cases.

## 4. Discussion

This study evaluated the diagnostic performance of two contemporary LLMs, ChatGPT-4o and Claude 3.5 Sonnet, for brain tumor MRI interpretation and compared their accuracy with that of radiologists across different experience levels, including the impact of LLM assistance on readers. Overall, while LLMs demonstrate promising potential as diagnostic support tools, human expertise remains indispensable.

Claude 3.5 Sonnet showed higher primary diagnosis accuracy and top-three differential diagnosis accuracy than ChatGPT-4o, although the differences were not statistically significant. Both LLMs were outperformed by board-certified radiologists in primary diagnosis accuracy, whereas their top-three differential diagnosis performance was comparable, highlighting their potential to generate clinically relevant diagnostic considerations. Notably, LLM assistance improved top-three differential diagnosis accuracy for both board-certified and trainee radiologists, and significantly increased trainee performance in primary diagnosis accuracy. Accordingly, we first discuss the educational and decision-support value of LLM assistance for trainees, followed by comparisons with expert readers and considerations for challenging tumor subtypes and workflow integration.

### 4.1. LLM-Assisted Improvement in Trainee Performance

One of the most significant and clinically relevant findings in our study is the enhanced diagnostic performance of resident radiologists when assisted by LLMs. Prior to assistance, trainees showed a primary diagnosis accuracy of 48.0%, which significantly increased to 58.8% (*p* < 0.001) after reviewing LLM generated suggestions. The improvement in top-three differential diagnosis accuracy was even more pronounced, rising from 62.5% to 81.1% (*p* < 0.001). This post-assistance accuracy of 81.1% essentially matched the baseline performance of the unassisted board-certified radiologists (80.7% in the top-three list). These results underscore the potential of LLMs to augment the diagnostic capabilities of less experienced clinicians, functioning as real-time educational and decision-support tools that provide on-demand insights and reinforce clinical reasoning.

This finding aligns with Horiuchi et al., who found that ChatGPT improved residents’ diagnostic accuracy by refining their differential lists, especially for nuanced or ambiguous cases [11]. Likewise, Ali et al. highlighted the role of LLMs as decision-support tools that assist clinicians, including residents, in clinical reasoning [1]. Srivastav et al. highlighted ChatGPT’s potential as an interactive educational platform offering real-time diagnostic feedback to trainees [15]. Khosravi et al. also underscored the importance of AI-assisted mentorship in radiology education, where simulated complex cases can enhance learning outcomes and diagnostic confidence [16]. Taken together, these findings suggest that LLMs, such as ChatGPT-4o and Claude 3.5 Sonnet, can play a significant role in radiology training, bridging the gap between novice and expert performance and improving diagnostic consistency in clinical practice.

### 4.2. Comparative Diagnostic Performance and Assistive Utility of LLMs

The superior primary diagnosis accuracy achieved by board-certified radiologists can be attributed to their extensive clinical experience and specialized training, which enable them to recognize subtle, often integrated, imaging features critical for precise brain tumor differentiation. This finding aligns with comparative studies consistently showing the advantage of human expertise over current-generation LLMs. For instance, Horiuchi et al. reported that LLM’s final diagnostic accuracy was lower than that of radiologists, particularly in cases requiring detailed analysis of intricate imaging features [11]. Similarly, Suh et al. found that while LLMs showed promise, radiologists consistently outperformed them, especially in identifying complex tumor subtypes [9]. Mitsuyama et al. likewise observed that, although LLM performed well in routine cases, it struggled in more complex scenarios where radiologists’ interpretive skills were critical [5]. These observations emphasize that while LLMs hold diagnostic promise, they currently lack the nuanced contextual understanding and robust clinical experience required for the most intricate cases, reinforcing the indispensable role of human radiologists.

Despite the advantages of human expertise, the comparable top-three differential diagnosis accuracy between the LLMs and board-certified radiologists in our study highlights the growing diagnostic potential of AI systems. This suggests that LLMs can leverage their broad medical knowledge to generate clinically relevant differential diagnoses based on complex imaging features. Mitsuyama et al. also found that ChatGPT-4, achieved a diagnostic accuracy of 73% when interpreting real-world radiology brain tumor reports, reinforcing its utility in generating useful differential diagnoses [5]. Additionally, Wada et al. demonstrated that prompt engineering strategies can significantly improve the diagnostic output of LLMs, particularly in generating top-three differential diagnoses, underscoring the potential of LLMs to augment diagnostic tasks [7]. Sarangi et al. further found that advanced LLMs such as Claude 3.5 Sonnet can assist radiologists in formulating comprehensive lists of potential diagnoses, even in challenging cases where rare entities might otherwise be overlooked [3]. In line with these previous findings, our study also demonstrated the practical benefit of LLM assistance. With LLM support, board-certified radiologists exhibited a significant improvement in top-three differential diagnostic accuracy, increasing from 80.7% to 90.2%. Taken together, these results suggest that LLM-assisted interpretation can help reduce diagnostic oversight and encourage consideration of a wider range of possible tumor types, especially in challenging cases.

### 4.3. LLMs in Complex Diagnostic Scenarios

LLMs demonstrated strong diagnostic performance for common brain tumor types; however, their accuracy decreased for rare or complex tumors. In our subgroup analysis, both Claude 3.5 Sonnet and ChatGPT-4o showed reduced performance in less frequently encountered tumor subtypes, which is consistent with prior reports indicating similar challenges in complex neuroradiological and CNS tumor settings [6,9,11,15]. Srivastav et al. noted that AI models trained on limited datasets may generate incomplete or outdated diagnostic suggestions, particularly for rare or atypical presentations [15]. This underscores a persistent gap: The subtle, nuanced imaging features characteristic of rare tumors—features often recognized only by experienced specialists—are not fully represented in current LLM training scopes.

In addition, atypical imaging presentations further contributed to diagnostic difficulty. In these scenarios, LLM-generated differential diagnoses may still provide clinical value by broadening the diagnostic consideration range, even when the primary diagnosis is incorrect. This aligns with recent findings by Nakaura et al. which reported markedly poor performance of LLMs in certain tumor subtypes, noting that Top 1 diagnostic accuracy for brain tumors, particularly for less common types such as CNS lymphoma with 0% accuracy, highlights the challenges LLMs in achieving the level of expertise exhibited by board-certified radiologists [12]. Consistent with their observations, our study also demonstrated a substantial decline in LLM accuracy for primary CNS lymphoma, with a primary diagnostic accuracy of only 40%. Atypical cases were especially challenging; for example, the case illustrated in Figure 1—a primary CNS lymphoma with unusual central necrosis—was not correctly diagnosed by either LLMs or human readers. These findings emphasize that both LLMs and radiologists may struggle when tumors present with uncommon or misleading imaging features, reinforcing the need for multimodal information and careful clinical integration, particularly for rare or atypically manifesting tumor types. This is supported by our findings that LLM assistance improved top-three differential diagnosis accuracy not only among trainees but also among board-certified radiologists, suggesting that LLMs can help reduce premature narrowing of diagnostic possibilities in challenging cases.

For rare or complex tumors, multimodal data integration is likely critical for advancing LLM diagnostic performance. In cases with potentially conflicting diagnostic indicators, LLM outputs appeared to rely primarily on structured imaging descriptors, whereas human readers may incorporate broader clinical context and experiential judgment. This distinction underscores the importance of integrating multidimensional clinical and imaging information to improve the robustness and clinical applicability of LLM-assisted diagnostic systems [17]. Doshi et al. and Wada et al. emphasized that incorporating complementary clinical information such as patient history, molecular profiles, and advanced imaging parameters can enhance reasoning and diagnostic precision [2,18]. To address these shortcomings, future development should focus on expanding LLM training datasets to include more diverse and multimodal imaging data, especially for rare and complex tumor types. Integrating clinical, genetic, and laboratory information could further improve diagnostic accuracy of LLMs, as suggested by Khosravi et al. and Ueda et al., who advocated for multimodal integration and continuous dataset updates to better handle complex neuro-oncologic scenarios [10,15]. The assistive benefit observed in our study may also reflect the structured diagnostic framework used during prompting, which encouraged systematic feature interpretation. McDuff et al. emphasized that fine-tuning LLMs with diverse datasets could improve their ability to generate accurate differential diagnoses, particularly for rare cases [19]. Rawas et al. further highlighted that continued algorithmic refinement will be necessary for LLMs to manage increasingly complex diagnostic tasks and to function effectively in real-time clinical workflows [20].

### 4.4. LLM Integration into Radiology Workflows

The potential role of LLMs in clinical radiology is substantial and multifaceted. As demonstrated in previous studies, LLMs can serve as decision-support tools, offering AI-generated differential diagnoses that may prompt further investigation or reconsideration of initial impressions. Nakaura et al. found that LLMs could reduce inter-radiologist variability, particularly in subjective interpretations influenced by cognitive biases [21]. Akinci et al. emphasized the value of LLMs in structured report generation, helping radiologists maintain consistency and minimizing omissions [22]. Such tools are especially valuable in resource-limited environments where access to subspecialty expertise may be constrained. In these contexts, LLMs can act as virtual second opinions, supporting radiologists in achieving more accurate and confident diagnoses. Future integration of quantitative neuroimaging biomarkers, including functional and network-level features, may further enhance the diagnostic capabilities and clinical utility of LLM-assisted radiologic interpretation [23].

Moreover, incorporating LLMs into radiology workflows has the potential to enhance diagnostic accuracy and efficiency, particularly for complex or ambiguous cases. Khosravi et al. emphasized that rigorous evaluation is essential to ensure AI systems meet the standards of clinical reliability required for patient care [16]. Akinci et al. further suggested that LLMs could streamline reporting processes and offer on-demand clinical insights [22], while Srivastav et al. stressed that LLMs should complement, not replace, human radiologists, enhancing precision and equity in healthcare delivery [15]. LLMs such as ChatGPT-4o and Claude 3.5 Sonnet could be integrated into clinical workflows as real-time decision support systems, helping radiologists manage complex diagnostic tasks and reduce variability in imaging interpretation.

However, it is important to acknowledge that the present study employed a snapshot-based approach using selected representative images rather than full volumetric sequences. Future studies should aim to incorporate volumetric imaging data and more interactive viewing environments when evaluating LLM-assisted diagnostic performance. The integration of volumetric imaging data into LLM-supported workflows may allow for more clinically realistic assessments and better alignment with routine radiologic practice.

## 5. Limitations

This study has several limitations. First, radiologists were not provided with the structured report form that was available to the LLMs, meaning that diagnostic performance was not assessed under identical information conditions. In addition, radiologists made diagnoses based on a single representative slide, which may have limited their ability to assess the full spectrum of imaging characteristics. Nonetheless, this study design reflects the current practical constraints of LLM-assisted interpretation, as most LLMs presently operate on selected key images rather than full volumetric MRI data. Thus, while this may underestimate radiologist performance, it also approximates a realistic LLM-use scenario. Furthermore, because the structured reports were generated by an experienced neuroradiologist based on review of the full MRI examination, some imaging descriptors may inherently correlate with specific tumor types. This introduces a potential risk of information leakage and limits the interpretation of the LLM as an independent diagnostic agent. Consequently, it remains difficult to determine whether the observed LLM performance primarily reflects analysis of visual information from representative images or the processing of structured feature-level inputs. Future studies comparing LLM performance with and without structured reports under identical imaging conditions would help clarify the relative contributions of visual and textual information.

Second, it remains difficult to determine whether the observed LLM performance was primarily derived from analysis of visual information in the representative slide or from processing the structured MRI reports provided by the expert neuroradiologist. Ideally, a comparative analysis evaluating LLM performance with and without structured reports under identical image conditions would help disentangle the relative contributions of visual versus textual input. However, because the present study employed a single run design and did not systematically assess output variability across repeated inferences, the reproducibility of LLM responses could not be fully evaluated. Future studies should incorporate reproducibility in LLM-assisted radiologic interpretation.

Third, this study did not formally evaluate potential cognitive effects of LLM assistance, such as anchoring bias, over-reliance, or reduced diagnostic exploration. Future investigations should incorporate study designs specifically aimed at assessing these cognitive and behavioral impacts.

Fourth, LLM outputs are inherently non-deterministic, and each case in this study was evaluated using a single inference per model. As a result, output variability and reproducibility across repeated runs could not be assessed. Future investigations should incorporate repeated inference strategies and consensus-based evaluation to better characterize the stability and reproducibility of LLM-assisted diagnostic performance. Statistically, a formal multi-reader multi-case (MRMC) analysis was not performed. Although five readers participated, the unbalanced dataset limited the feasibility of implementing a fully crossed MRMC framework. Consequently, reader–case clustering effects were not explicitly modeled, and the statistical comparisons may not fully account for inter-reader variability. Future studies incorporating complete reader participation and balanced datasets will be necessary to enable robust MRMC-based statistical analysis.

Fifth, the dataset included only primary brain tumors, whereas brain metastasis is the most common intracranial neoplasm in adults [24]. Because the dataset lacked clinical information regarding systemic malignancy, both radiologists and LLMs occasionally misclassified primary tumors as metastatic lesions. In real clinical practice, however, the diagnostic approach to metastasis relies heavily on clinical context, including the presence or absence of known primary cancer and the multiplicity of intracranial lesions. Therefore, the results of this study should be interpreted separately from real-world diagnostic conditions in which such clinical information is routinely integrated.

Sixth, we did not include other commercially available LLMs such as Google Gemini. The LLMs evaluated in this study were among the most up-to-date versions at the time of analysis. However, LLMs evolve rapidly with frequent model updates. Therefore, we cannot make a fair comparison with newer or more advanced Gemini versions released after our study period.

Finally, the ethical and legal implications of LLM deployment in clinical practice require careful consideration. Key issues include responsibility in the event of diagnostic error, data privacy, and the potential for inequitable access or amplification of healthcare disparities. Prior studies have emphasized the need for clear regulatory frameworks, continuous model validation, and training strategies that support radiologists in effectively supervising and integrating AI systems into diagnostic workflows [12,18,25]. Ensuring safe and equitable implementation will be essential as LLM-based tools evolve.

## 6. Conclusions

In this cohort of 127 pathologically proven brain tumors, contemporary LLMs, specifically ChatGPT-4o and Claude 3.5 Sonnet, showed the potential to broaden differential diagnostic considerations when operating on structured imaging inputs. Within the constrained, snapshot-based experimental setting of this study, LLM assistance was associated with improvements in trainee diagnostic performance. However, these findings should be interpreted cautiously, as the study design involved structured feature inputs and limited imaging representation. Further studies using balanced input conditions, full volumetric imaging workflows, and robust multi-reader statistical frameworks are needed to more comprehensively evaluate the reliability and clinical applicability of LLM-assisted radiologic interpretation.

## Figures and Tables

**Table 1:** Pathologic Classification and Distribution of Brain Tumors.

Tumor Types	No. (Male)	Age (Mean)
Astrocytoma	15 (10)	42.7
Astrocytoma, Gr 2	6 (4)	39.0
Astrocytoma, Gr 3	5 (4)	43.8
Astrocytoma, Gr 4	4 (2)	46.5
Glioblastoma	15 (8)	65.3
Oligodendroglioma	15 (7)	51.8
Oligodendroglioma, Gr 2	7 (3)	50.3
Oligodendroglioma, Gr 3	8 (4)	53.1
Hemangioblastoma	15 (6)	53.6
Primary CNS lymphoma	15 (6)	71.0
Medulloblastoma	10 (8)	12.4
Pilocytic astrocytoma	10 (4)	38.6
Ependymoma	10 (3)	30.9
Ganglioglioma	8 (3)	22.1
DNET	7 (4)	13.4
Diffuse midline glioma	7 (3)	34.8

**Table 2:** Comparison of diagnostic accuracy between Claude 3.5 Sonnet and Radiologists.

Group	Primary Diagnosis Accuracy (95% CI)	Top-Three Diagnosis Accuracy (95% CI)
Claude 3.5 Sonnet	50.39% (41.6–59.1%)	85.04% (77.6–90.5%)
Board-certified Radiologists	69.3% (60.8–76.6%)	80.7% (72.6–86.3%)
Trainee Radiologists	48.0% (39.5–56.7%)	62.5% (53.5–70.2%)

**Table 3:** Changes in radiologists’ diagnostic accuracy with LLM Assistance.

Group	Metric	Baseline (95% CI)	After LLM Assistance (95% CI)	*p*-Value
Board-certified Radiologists	Primary diagnosis	69.3% (60.8–76.6%)	71.3% (62.4–78.1%)	0.166
	Top-three diagnosis	80.7% (72.6–86.3%)	90.2% (83.3–93.9%)	<0.001
Trainee Radiologists	Primary diagnosis	48.0% (39.5–56.7%)	58.8% (49.6–66.5%)	<0.001
	Top-three diagnosis	62.5% (53.5–70.2%)	81.1% (72.6–86.3%)	<0.001

## Data Availability

The data that support the findings of this study are available from the corresponding author, upon reasonable request.

## Abbreviations

The following abbreviations are used in this manuscript:

AI	Artificial intelligence
CNS	Central nervous system
DNET	Dysembryoplastic neuroepithelial tumor
DWI	Diffusion-weighted imaging
FLAIR	Fluid-attenuated inversion recovery
LLM	Large language model
MRI	Magnetic resonance imaging
PWI	Perfusion-weighted imaging
SWI	Susceptibility-weighted imaging
